# Universal Screening for Firearm Injury Risk Could Reduce Healthcare’s Hesitancy in Talking to Patients About Firearm Safety

**DOI:** 10.1097/AS9.0000000000000121

**Published:** 2022-01-10

**Authors:** Chethan Sathya, Sandeep Kapoor

**Affiliations:** From the * Division of Pediatric Surgery, Cohen Children’s Medical Center, Northwell Health, New Hyde Park, NY; † Donald and Barbara Zucker School of Medicine at Hofstra/Northwell, Hempstead, NY; ‡ Center for Gun Violence Prevention, Northwell Health, New Hyde Park, NY.

Despite vast long-standing healthcare industry support for a public health approach to firearm injury and mortality prevention—*still*—the minority of physicians, surgeons, nurses, and social workers ask patients questions about firearm access or gun violence risk, let alone counsel about firearm safety.^1,2^ For most of us, screening and counseling related to firearm injury prevention, although demonstrated to be beneficial,^3^ continues to fall outside the umbrella of “usual care” and is often reserved for patients deemed “high risk” such as those presenting with suicidality, assault-related injuries, or perceived risk.^1–3^

But for us to practice what we preach, a complete rethink using a novel, unconventional, and nondiscriminatory universal firearm injury risk screening approach across trauma, emergency, and primary care settings—for all patients regardless of reason for visit—may be the transformative change needed to shift the paradigm. By asking each and every patient questions about firearm injury risk as part of routine surgical or medical care, we may finally be able to break down barriers and get comfortable talking to patients about firearm safety. After all, for any public health issue, it all starts with surveillance and screening. If we cannot get comfortable asking the necessary questions in the first place, holistic progress from the healthcare lane on our nation’s public health crisis of firearm injury and violence will continue to prove elusive.

A “we ask everyone” approach has been used successfully in dealing with other polarized public health issues, such as HIV, tobacco use, and substance use,^4^ suggesting that it could be beneficial in normalizing and humanizing conversations about firearm safety. A universal approach can effectively remove the decision point for clinical team members of whether to screen or not, thereby avoiding stigmatization, judgment, and unnecessary profiling of patients based on risk perception.^4^ As one can imagine, this could significantly increase comfort with this topic from both the healthcare worker and patient perspective. And virtually all health care providers, including those in trauma, emergency, geriatric, pediatric, and primary care settings, are uniquely poised to integrate these questions into existing routine injury prevention guidance. So why not consider the same approach for firearm injury prevention?

Undeniably, targeted screening for firearm injury risk is far more feasible than a universal approach, especially considering that healthcare workers already have numerous risk factor screening questions to ask.^1,2^ Many would argue that if we ask everyone about firearm safety, we could overburden clinical staff with questions that may not yield a significant number of “positive” screens. Yet firearm injury and mortality remains one of the leading causes of preventable death in the United States.^1^ According to a recent Centers for Disease Control report, 39,000 Americans died from firearm injury in 2018, let alone countless others who suffered trauma and morbidity from nonfatal firearm injuries. Gun violence is nothing short of endemic and its prevalence alone demands that we creatively use all resources available to address this public health issue.^1^ Hence, implementing a universal approach to screening and intervening among those at risk of firearm injury is certainly warranted and overdue, and will allow us to perform research in parallel to identify barriers and determine what works and what does not.

Due to the paucity of research on firearm injury preventative strategies, resulting from decades of limited research funding, we must not lose sight of the fact that in many ways firearm injury is akin to a new disease. We lack the data to completely understand who is “at risk” and how levels of firearm injury risk may vary in different populations.^1,2^ Hence, by targeting our questions, we potentially miss out on screening cohorts of patients who may be at risk.^4^ We also risk stigmatizing the conversation and further impeding our ability to normalize the conversation of firearm injury prevention with our patients—*ALL* our patients. In doing so, we move farther away from one of our ultimate goals—which is to shift the paradigm in the healthcare industry to consider questions related to firearm safety as part of routine care, no different than the standard questions we ask patients pertaining to fall risk, sugar intake, smoking, substance use, and seat belt use. With these risk factors, we readily partner to provide respective guidance in an effort to support patient and community well-being.

To be clear, when it comes to physicians, surgeons, nurses, social workers, or any healthcare workers asking patients questions about firearm access and gun violence risk—the motivation is to promote firearm safety and prevent firearm injury and mortality. The majority of patients, gun-owning or not, support their physicians in asking these questions as they understand it relates to safety.^3^ In addition, we know that clinical team member counseling on firearm safety can save lives and that particular strategies, such as hospital-based violence intervention programs, are essential to a holistic approach to gun violence prevention.^2^ So why after decades is uptake of firearm injury risk screening, counseling, and other healthcare-driven harm reduction strategies still so limited?

The simple answer is—we don’t know. We have not researched preventative strategies enough to fully understand their effectiveness, let alone the facilitators and barriers to implementation.^1–3^ With recently awarded federal funding for firearm injury prevention research, however, comes a wave of legitimacy toward studying and understanding firearm injury. This research will undoubtedly provide us the data needed to approach gun violence as a public health issue, similarly to solutions that were developed for substance use. In the meantime, it would be prudent for us to do our due diligence in comprehensively researching the basics of this public health issue, while concurrently implementing strategies supported by early data.

Based on the limited studies evaluating healthcare worker hesitancy on this issue, there are a number of barriers that likely play a role in why so few clinical team members talk to their patients about firearms.^1,2,5^ These include a lack of education on how to ask or counsel, concern for appearing judgmental or harming the doctor-patient relationship, an incomplete understanding of the legality, and inadequate infrastructure to provide patients with the resources needed as part of the intervention.^5^ Also, limited time is a big issue for healthcare workers who are inundated with risk factor screening. Hence, a team-based approach including a variety of clinical team members, such as physicians, nurses, social workers, and community organizations, is required and could significantly improve feasibility and uptake.^4,5^

Though arduous in process, we must take a step back and rigorously evaluate how we can improve clinical team member and patient comfort discussing this sensitive topic, as well as address barriers to implementation. Doing so will pay dividends in allowing us to normalize conversations about firearm safety between healthcare workers and patients. A universal “we ask everyone” approach to screening and intervening to identify those at risk of firearm injury is an important first step in integrating questions related to firearm safety into medical vernacular as part of “routine care” and shifting the mentality of the healthcare industry to view these questions as no different than asking about other modifiable risk factors. Only then—will we really move the needle forward on this devastating public health crisis.
